# Cardiovascular Effects of Cosmic Radiation and Microgravity

**DOI:** 10.3390/jcm13020520

**Published:** 2024-01-17

**Authors:** Omar Giacinto, Mario Lusini, Emanuele Sammartini, Alessandro Minati, Ciro Mastroianni, Antonio Nenna, Giuseppe Pascarella, Davide Sammartini, Massimiliano Carassiti, Fabio Miraldi, Massimo Chello, Francesco Pelliccia

**Affiliations:** 1 Research Unit of Cardiac Surgery, Department of Cardiovascular Surgery, University Campus Bio-Medico, 00128 Rome, Italy; 2 Centro per la Lotta Contro L’Infarto, 00182 Rome, Italy; 3 Department of Cardiovascular Sciences, Università Sapienza, 00185 Rome, Italy; 4 Research Unit of Anaesthesia and Intensive Care, Department of Medicine, University Campus Bio-Medico, 00128 Rome, Italy

**Keywords:** cosmic radiation, electromagnetic spectrum, microgravity, prevention therapies, radiation-induced DNA damage, spaceflights, solar radiation

## Abstract

Recent spaceflights involving nonprofessional people have opened the doors to the suborbital space tourism business. However, they have also drawn public attention to the safety and hazards associated with space travel. Unfortunately, space travel involves a myriad of health risks for people, ranging from DNA damage caused by radiation exposure to the hemodynamic changes that occur when living in microgravity. In fact, the primary pathogenetic role is attributed to cosmic radiation, since deep space lacks the protective benefit of Earth’s magnetic shielding. The second risk factor for space-induced pathologies is microgravity, which may affect organ function and cause a different distribution of fluid inside the human body. Both cosmic radiation and microgravity may lead to the alteration of cellular homeostasis and molecular changes in cell function. These, in turn, might have a direct impact on heart function and structure. The aim of this review is to draw attention to the fact that spaceflights constitute a novel frontier in biomedical research. We summarize the most important clinical and experimental evidence regarding the cardiovascular effects of cosmic radiation and microgravity. Finally, we highlight that unraveling the mechanisms underlying how space radiation and microgravity affect the cardiovascular system is crucial for identifying potential countermeasures and developing effective therapeutic strategies.

## 1. Introduction

In July 2021, Richard Branson and Jeff Bezos, the two billionaire founders of the spaceflight companies Virgin Galactic and Blue Origin, launched into suborbital space and had the privilege of experiencing a brief period of weightlessness and enjoying an extraordinary view of Earth against the blackness of space. Notably, Branson was able to cross the Kármán line, the boundary between Earth’s atmosphere and outer space, which is located 62 miles (100 km) above the ground. These two spaceflights opened the doors to the suborbital space tourism business but, at the same time, brought public attention to the safety and hazards of space travel. These hazards include the effects of different gravitational forces, the physical and psychological consequences of living isolated in closed environments, and, most importantly, exposure to two dangerous factors that are associated with health risks, i.e., cosmic radiation and microgravity. There is now evidence that these two factors play a special role in the development of acute and chronic diseases during manned missions [1]. Although there is much work that needs to be done, it is now clear that space exploration has the potential to cause multiple short-term and long-term complications, as it can affect any or all of the six organ systems, i.e., the respiratory, renal, hepatic, hematologic, cardiovascular, and neurologic systems [2].

In this review, we aim to call attention to the fact that spaceflights constitute a novel frontier in cardiovascular research. To this end, we summarize the most important clinical and experimental works showing the effects of cosmic radiation and microgravity on the cardiovascular system. Also, we highlight that unraveling the mechanisms underlying how space radiation and microgravity affect the cardiovascular system is crucial for identifying potential countermeasures and developing ad hoc prevention and therapeutic approaches.

## 2. Cosmic Radiation

**Figure 1:**
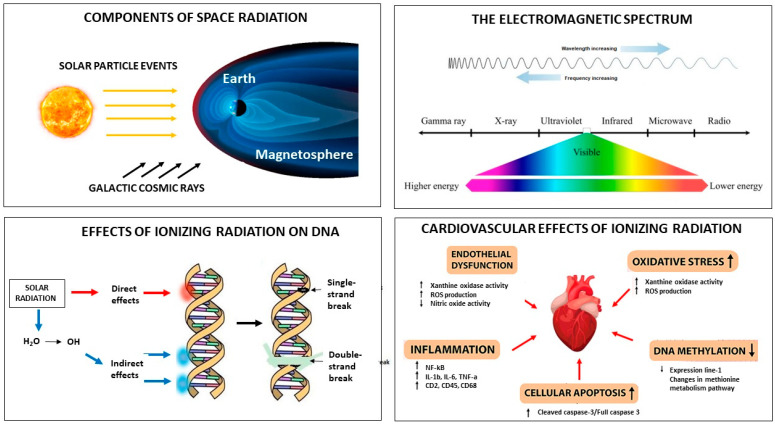
Space radiation. The components of solar radiation include galactic cosmic rays and solar particle events (**upper left**). The electromagnetic spectrum shows that cosmic rays are waves with the highest frequency and the shortest wavelength (**upper right**). Solar radiation may produce changes in DNA either directly via direct energy absorption or indirectly via the production of reactive oxygen species (**lower left**). The cardiovascular effects of solar radiation include endothelial dysfunction, increased oxidative stress, inflammation, increased cellular apoptosis, and decreased DNA methylation (**lower right**).

The radiation environment of deep space is very different from that at Earth’s surface or in low-Earth orbit. Earth’s magnetosphere protects us from harmful space energy, but when outside our planet, space travelers are exposed to low-dose-rate galactic cosmic rays (GCRs) and intermittent solar particle events (SPEs) (Figure 1) [3]. GCRs originate from outside the solar system and are high-energy protons and atomic nuclei that move through space almost at the speed of light. Within the spectrum of GCRs, the so-called HZE ions (high (H) charge (Z) and energy (E) ions) may cause more severe biologic injury than protons and particles. Therefore, they differ significantly from other forms of radiation (i.e., X-rays and gamma-rays) (GRAPHICAL ABSTRACT). Luckily, the magnetosphere protects the solar system from GCRs, which are affected by Lorentz forces and consequently pushed back by solar wind. This explains why no relevant long-term problems have been recorded in astronauts orbiting around Earth in the International Space Station. 

SPEs constitute the other part of space radiation. SPEs occur when particles emitted by the Sun, mostly protons, become accelerated either in the solar atmosphere during a solar flare or in interplanetary space. Of note, the doses of SPE that one is exposed to while in space are greater than those encountered on Earth (e.g., around 3 mSv per year) [3].

A large body of experimental data has elucidated the ultrastructural effects of exposure to space radiation in recent years [4]. Although clinical studies are needed to fully understand the consequences of space exploration in humans, available in vivo information indicates that the mechanism involved in the radiation-induced damage of DNA, RNA, and proteins is twofold: direct damage is caused by direct energy absorption and indirect injury is caused by the radiolysis of water molecules, which produces reactive oxygen species (ROS) (Figure 1) [5]. The amount of radiation-induced biological damage depends either on the total dose of radiation or on the type of radiation. Any given type of radiation is associated with a specific absorbed dose, which is the amount of energy absorbed per unit mass of the irradiated material. This concept has been termed “linear energy transfer” (LET) and is defined in terms of the energy lost per unit path length by the particles [5]. On Earth, the human body is exposed to low LET radiation (x radiation, beta radiation, or gamma radiation). Conversely, in space, high LET radiation affects the human body much more than low LET radiation because it can produce clusters of ions and radical particles. Specifically, high LET can cause genomic lesions when radiation passes through DNA. If not repaired, these single- or double-strand breaks can result in multiple damages, including point mutations and chromosome abnormalities, as well as carcinogenesis and apoptosis (Figure 1) [5].

As the space tourism business might offer long-distance space travel in the near future, having a full understanding of the health risks of space radiation is mandatory. Indeed, available evidence raises the concern that space radiation should be regarded as a previously unrecognized cardiovascular risk factor. Recent epidemiological studies, either in survivors of atomic bombs or in subjects exposed to low-dose radiation, indicate that the cardiovascular system is more prone than other organs to developing ionizing radiation damage. An important example is that Apollo lunar astronauts experienced significantly greater cardiovascular mortality than astronauts who did not fly beyond low Earth orbit. Lunar flight astronauts also had higher cardiovascular mortality compared to age-matched normal subjects, suggesting that short-duration deep space travel might result in an increased risk of cardiovascular death [6].

The pathophysiology of cardiovascular changes induced by space radiation has now been elucidated in-depth (Figure 1). Irradiation of cardiac tissue causes apoptotic cell death in several cardiac structures, such as vascular cells, cardiac myocytes, and fibroblasts [7]. Several experimental models have demonstrated the development of myocardial remodeling or fibrosis after exposure to HZE ions, such as protons or heavy ions, but not after exposure to gamma radiation. These findings indicate that 56Fe ions, one of the most prominent components of space radiation, are a major determinant of cardiac abnormalities and atherosclerosis [8]. Indeed, experimental investigations have shown that exposure of the heart to the heavy ions present in space radiation causes the release of cytokines and other molecules that have a pro-inflammatory effect, which, in turn, causes apoptotic cell death. Another mechanism involved in radiation-induced cellular damage is oxidative stress. Recently, investigators have also focused on the role of DNA methylation, which plays a major role in cellular homeostasis, as space radiation might affect the cardiovascular system through alterations in multiple components of the genome. Last but not least, one should take into consideration the fact that the integrity of the endothelium is crucial, as any vascular change has pro-inflammatory and pro-fibrogenic consequences. In particular, experimental work has clearly shown that space radiation exposure causes microvascular damage and endothelial dysfunction, which play a role in determining cardiovascular disease [9]. 

## 3. Microgravity

Bodies in the space environment experience a decrease in the Earth’s mass-relative gravity—a phenomenon known as microgravity. This means that the gravity vector decreases up to a certain point, but without reaching a complete absence of gravity because the mass of the body, even if small, plays a role in generating some gravity [10]. Conventionally, the absence of gravity is only achieved when the body is far enough from Earth’s surface to not be subject to the influence of Earth’s gravity. This phenomenon does not occur with vehicles orbiting Earth. In this situation, gravity cannot be completely canceled out. The overall effect that the body perceives as the absence of gravity is the result of the action of a weak gravitational force emanating from Earth’s mass and the counteracting centrifugal force to which the vehicle is subjected. If weightlessness is considered one of the biggest problems for astronauts in space due to the severe restriction of movement and the high speeds reached during work activities, the microgravity generated in this environment affects the physical response and leads to a number of potentially dangerous changes that can significantly impact the adaptation of the cardiovascular system. Current knowledge on the molecular and physiological changes that occur in the cardiovascular system after exposure to microgravity has been elucidated in several experimental studies (Table 1), with major findings as follows [11,12,13,14,15,16,17,18,19,20,21,22,23,24,25,26,27,28,29,30,31,32,33,34]. 

Spaceflight cardiovascular system clinics have been well-established since the first voyage around the globe in the 1960s. The absence of 1 g gravity significantly impacts blood distribution inside the body. In microgravity, blood peripheral storage is reduced and upper liquid sequestration is common. One of the most picturesque features of astronauts during space activities is the characteristic puffy face caused by the sequestration of liquid in soft tissues. This fluid rearrangement is particularly important for heart filling chambers. A decreased left ventricle diastolic volume may lead to a lower ejection fraction than in a ground position. This is of major concern during long space trips. Decreased left ventricular ejection fraction and stroke volume can induce a depression of inotropism and heart atrophy. This is the baseline feature of the parasympathetic syndrome that impacts astronauts upon returning to Earth. Noradrenaline and adrenaline activation is not enough to counteract peripheral vasodilation, resulting in rapid hypotension with difficulty in maintaining an orthostatic position. We should imagine the potential implications of this problem in deep space travels. A voyage to Mars would involve long periods of microgravity exposure. Although body exercises should be continuously performed, we do not know how cardiac muscle fibers may react to this prolonged environmental stress. Gathering the main studies on this topic may motivate researchers to describe the mechanisms of heart impairment after exposure to microgravity. As described earlier, many molecular pathways are involved in this pathophysiology. It would be an investigative upgrade to explore a common pathway that leads to the heart ejection fraction depression observed during the aforementioned studies. 

Genetical alterations are the main cause of the reduced expression of demethylation enzymes, leading to increased DNA methylation. DNA methyltransferase-1 and methylcytosine dioxygenase ten–eleven translocation-1 (TET1) are decreased, along with a reduction in the mRNA expression of alfa-1-actin [30]. This genetical modification leads to myocardial fibers with low inotropism. This first molecular step has to be considered alongside other environmentally induced alterations in biomolecular systems. The central core of molecular dysfunction may be represented by the formation of reactive oxygen species (ROS), the impairment of calcium intake, and the alteration of myocardial fibers. 

Microgravity plays a main role in the downregulation of the AC/cAMP system, particularly in its post-receptor response to stimulation. In the study conducted by Lokter et al. [30], isoproterenole/forskolin stimulation decreased activation of the post-receptor response with lower cAMP synthesis and secondary calcium channel dysfunction. Concomitant phosphorylation led to decreased intracellular calcium concentrations. Changes in cytosolic ion features caused an abnormality in the structure of myocardial tissue, evident in a shift from alpha-MHC to beta-MHC. This tissue change, along with the slower action of ATPase, is an etiological factor for the prolongation of atrophic fibers with secondary evidence of cardiac arrhythmias, a phenomenon recorded in astronauts during prolonged exposure.

Calpain activation is also important for cytosolic calcium concentration [32]. Calpain-2 may induce a temporary increase in the level of this ion, with the potential for myocyte apoptosis. The increased level of calcium inside the cell triggers the action of CaMKII, consequently phosphorylating S2814 on RyR2, leading to the activation of MEF-2 and the elevation of serum levels of ANP and BNP. This biochemical chain leads to cardiac remodeling with depression of the left ventricular ejection fraction and stroke volume. Cardiac function impairment is achieved by calpain, which triggers, in association with Atrogin-1, ERK2-1. ERK2-1 plays a role in the phosphorylation of Ser-345 on p47^phox^. The activation of p47 is a main factor in the formation of ROS, the expression of NF-kB, and the depression of IKBS and their inhibitory action on the synthesis of NF-kB1 (p50), NF-kB2 (p52), Rel A (p65), Rel B, and c-Rel. All of these molecular alterations lead to myocardial atrophy.

The alteration of the actin/myosin complex, as a consequence of the fibers shifting from alpha-MHC to beta-MHC, leads to an increase in titin concentration [31]. This protein is relevant in compensatory reactions to the depression of inotropism caused by alterations in the actin/myosin complex. In the microgravity environment, titin has an abnormal secondary structure, leading to a decrease in actin/myosin ATPase. Simultaneously, an increase in gamma-actin, in conjunction with a reduction in ATPase, contributes to the atrophy process.

Briefly, all the above-mentioned molecular alterations characterize the clinical aspects related to spaceflights. In particular, changes in the regulation of intracellular calcium ions vary with the duration of the flight, leading to obvious consequences during long trips. Myocardial atrophy, a common consequence of specific molecular impairments, should be matched with the structural damage related to microgravity exposure. To date, myocardial atrophy has only been studied as a prognostic factor in patients with reduced left ventricular ejection fraction and/or heart failure. There is a need for an assessment of valve competence after the remodeling processes of the heart and a direct investigation into the implications of long-lasting damage to the intima of coronary vessels, which potentially causes coronary artery disease.

Microgravity has been investigated as a factor influencing the gene expression of biomolecules. Methylation on the so-called cytosine followed by guanine residue islands (CpG) of DNA plays a main role in the regulation of gene expression. Recently, this particular aspect was the focus of a randomized trial involving groups of mice exposed to hindlimb suspension [31]. The groups included in the study were subjected to 30 days of microgravity simulation. No changes were detected in the methylation of CpG islands, resulting in normal gene expression of beta-actin, gamma-actin, alfa-actinin-4, and beta tubulin. The mRNA level of alfa-actinin-1 was lower than that of the control group, and this aspect was associated with an increase in CpG methylation. However, this value returned to normal after 12 h of being in the physiological rat position. An increased level of CpG methylation caused the partial suppression of desmine expression. Also, the enzymes involved in methylation/demethylation processes were tested. DNA methyltransferase-1 (DNMT-1) and the encoding mRNA were significantly decreased after 30 days of microgravity simulation, but were at the same level after 12 h of recovery. The reduced capacity of the demethylation process was characterized by a depression in methylcytosine dioxygenase ten–eleven translocation-1 (TET1), which appeared to be less represented after 30 days of hindlimb suspension. This reduction also remained constant after 12 h of recovery. Histone acetyl transferase-1 (HAT-1) was not affected by microgravity, whereas histone deacetylase-1 (HDCA-1) was upregulated in the microgravity simulation group. 

To investigate the impact of microgravity on cardiogenesis, an experiment was conducted on the Tianzhou-1 spaceflight [28]. Induced pluripotent stem cells (iPSCs) were positioned inside a bioreactor system and Oct4 green fluorescent proteins (Oct4-GFPs) and alpha-myosin heavy chain green fluorescent proteins (alphaMHC-GFPs) were tested. The size of embryoid bodies (EBs) increased during the first 4 days and remained constant for the entire experimental period (14 days). There was a peculiar decrease in the level of Oct4-GFP during the first 3 days. The upper and regular expression of alpha-MHC-GFP during the 14-day experiment demonstrated efficient cardiomyocyte differentiation.

In 2000, a significant study was performed on avian embryos regarding their myocardial mechanical development in microgravity [15]. In order to assess this, a rotated bioreactor was employed. In particular, the fibronectin (FN) and desmosomes involved in myocardial contraction were considered. FN is an extracellular matrix (ECM) glycoprotein, which has a role in primordial heart formation. It is located between precardiac mesoderm and endoderm. Inhibition of the gene that encodes FN plays a role in decreasing mesodermal migration and differentiation. A decreased level of FN in embryos results in reduced contractile capacity of the myocardium. Desmosomes have a particular role in chemical communication between myofibrils because they control gap junctions between the cells. The study mentioned above reported decreased contractility in embryos exposed to microgravity, as the reduction in FN and desmosomes is associated with this important aspect of heart mechanics. Another aspect underlined by the authors was the alteration of myofibril displacement caused by variability in acceleration due to the bioreactor force. The geometrical impairment of this structure led to an impairment in myocardial contraction.

In summary, molecular patterns play meaningful roles in determining the low performance of the heart caused by a reduction in left ventricular ejection fraction, especially after long spaceflights. Microgravity is the main risk factor for this disease. In recent years, this problem has emerged with a significant impact, given the new space programs scheduled by national space agencies worldwide. New interest in manned crew missions to the Moon, involving longer stays in orbit, poses a challenge for understanding the impact of the environment on the body, particularly on the cardiovascular system. As described, hemodynamic storms develop during prolonged stays in space and manifest when crew members return to Earth’s surface with 1 g gravity. Focusing on common molecular and biochemical alterations may, in the future, facilitate a pharmacological and/or molecular approach to interrupt the chain that leads to cardiovascular syndrome, particularly evident after landing on Earth. 

## 4. Therapeutic Implications

Humans operating in extreme environments often conduct their operations at the limits of human performance [35]. Unraveling the mechanisms underlying how space radiation and microgravity affects the cardiovascular system allows for the definition of potential countermeasures [36]. With respect to this, it is important to note that there is huge variability in the needs of those engaged in extreme operations because of differences in the ability to match each individual’s specific molecular phenotype with any given environment. Since space radiation and microgravity play variable roles in influencing the CVS in the space environment, the development of personalized pharmacological therapeutic strategies would be noteworthy. Currently, there are no large studies that provide evidence for such therapies.

With regards to radiation, current shielding technology, which would be viable for use in spacecrafts, does not seem capable of eliminating radiation risk [37,38]. Conversely, the preventive use of multiple cardiovascular drugs, including anti-cholesterol, anti-inflammatory, antihypertensive, and metabolic agents, has the potential to limit radiation damage from space voyages [9,10]. By inhibiting the 3-hydroxy-3-methyl-glutaryl-coenzyme A reductase, statins may be beneficial for astronauts, as they have anti-neoplastic properties by activating pathways with caspases 3 and 9 and anti-inflammatory effects by inhibiting the upregulation of E-selectin and neutrophil chemotaxis. Experimental work has shown that statins decrease vascular changes after radiation exposure, as they protect endothelial cells from irradiation [9,10]. Similar to statins, non-steroidal anti-inflammatory drugs can exert protective effects. The cyclooxigenase-2 inhibitor meloxicam decreases 30-day mortality in mice in 9 Gy whole-body irradiation when given 1 h before or after exposure to radiation. Angiotensin converting enzyme inhibitors (ACE-Is) and angiotensin receptor blockers (ARBs) may reduce radiation-induced damage to the cardiopulmonary system by increasing free radical scavenging and reducing lipid peroxidation and DNA alteration [9]. However, the potential side effects of ACE-Is and ARBs, such as decreased renal perfusion and angioedema, limit their use. Indeed, the venous blood pooling occurring during spaceflight due to microgravity in low Earth orbit or the absence of gravity in deep space voyages might predispose to the formation of edema caused by the increased levels of bradykinin induced by ACE-Is and ARBs. New preventive strategies include N-acetylcysteine, a thiol-containing antioxidant that may scavenge free radicals and decrease levels of caspase-3, and metformin, which may protect from damage caused by reactive oxygen species, thus increasing the survival of fibroblasts and endothelial cells after exposure to radiation. Calcium channel blockers inhibit low-density lipoprotein oxidation at high concentrations and have a direct cellular protective effect at lower concentrations, which mitigates the vascular changes induced by oxidative stress in atherosclerosis. 

With regards to microgravity, the role of propranolol treatment in limiting the formation of oxygen radicals and scavenging them has been postulated [39]. A similar effect has been suspected in radiation exposure and could result in the formation of oxygen radicals in response to microgravity. Given its pharmacodynamic and pharmacokinetic effects, which depend on its role as an inhibitor of xanthine oxidase and superoxide formation [40], propranolol could be introduced as a prophylactic measure, along with omega-3 polyunsaturated fatty acids and vitamins C and E. Because oxidative stress is so closely linked to the microgravity environment, molecules from the diet can be used to counteract the effects of oxygen free radicals. Nitrate and nitrite, found in green vegetables, and the precursors of NO lycopene, found in tomatoes, can be included in the diet to induce a preventative antioxidant effect [41]. Other molecules, such as nicotinamide riboside, which is found in milk and yeast foods, contribute to the formation of oxidized nicotinamide adenine dinucleotide. This molecule activates the nicotinamide-adenine-dinucleotide-dependent protein deacetylase chain sirtuin 1, which triggers the production of NO. It also plays a role in scavenging oxygen free radicals [42]. 

## 5. Conclusions

Space tourism is a new segment of the aviation industry that is going to allow the general public to experience travel outside Earth for recreational leisure. Unfortunately, space travel involves a myriad of health risks for people, ranging from DNA damage caused by radiation exposure to hemodynamic changes that occur when living in microgravity. Similarly, spaceflights constitute a new frontier in biomedical research. A marked new interest in this field has developed since the first missions around the globe and the Apollo program. Over the last two decades, the laboratory department inside the International Space Station (ISS) has allowed experiments to be conducted in low Earth orbit (LEO). The evaluation of physiological behaviors in space is crucial, as several space missions have been scheduled over the next few years. Interestingly, a new approach to Moon exploration has been planned by government and private space agencies. This new interest in our satellite has arisen from the possibility of reaching Mars. For this reason, the Moon may now be used as a training place for longer trips and a place where experiments in deep space can be carried out. Currently, the scientific community is aware of the environmental risks that space crews may be exposed to. Cosmic radiation plays a crucial role, as in deep space, it is not possible to benefit from magnetic Earth shielding. Fare clic o toccare qui per immettere il testo. The second risk factor for space-induced pathologies is microgravity, which may affect organ function and cause a different distribution of fluid inside the human body. Microgravity has another important role in altering cellular homeostasis and cell function. Exposure to lower gravity forces has a direct impact on heart function and structure. Considering the long-planned voyages and extended durations of stay on the Moon and, eventually, on Mars, it is mandatory to consider microgravity as an independent factor for cardiovascular disease. 

In conclusion, there is now clear evidence that space exploration plays a special role in the development of acute and chronic diseases [1]. However, further investigations are needed in order to define the extent to which cardiovascular risk is increased by cosmic rays and microgravity, to fully elucidate the pathophysiology of damage induced by space exploration, and to identify novel options to manage and possibly prevent radiation- and microgravity-induced cardiovascular conditions.

## Figures and Tables

**Table 1:** Summary of the most relevant experimental studies on microgravity to date.

Author	Year	Method	Results
Mednieks et al. [11]	1986	Rat model (retrospective)	Decreased K_m_ cAMP phosphodiesterase
Philpott et al. [12]	1990	Rat model (retrospective)	Mitochondrial degeneration, decreased ATP, ischemic damage
Fareh et al. [13]	1994	Rat model (retrospective)	Increased irANP
Connor et al. [14]	1998	Rat model, spaceflight (retrospective)	Increased MDH, subunit IV, decreased GAPDH
Lwigale et al. [15]	2000	Avian embryos (retrospective)	Decreased FN and desmosomes
Yu et al. [16]	2001	Rat model (retrospective)	TM fragments
Okumura et al. [17]	2007	Rat model (retrospective)	AC expression
Yin et al. [18]	2008	Rat model (retrospective)	SC/cAMP depression
Kwon et al. [19]	2009	Rat model (retrospective)	Upregulation of NF-kB
Ito et al. [20]	2010	Rat model (review)	Decreased SERCA2a/PLB ratio
Cui et al. [21]	2010	Rat model (retrospective)	Ca^2+^ channel dysfunction
Chang et al. [22]	2011	Rat model (retrospective)	Calpain induced apoptosis
Vykhlyantsev et al. [23]	2011	Meriones unguiculatus model, spaceflight (retrospective)	Titin action and structure
Ogneva et al. [24]	2012	Rat model (retrospective)	Myocardial stiffness/beta, gamma, alfa-actinin-1 and alfa actinin-4
Schwoerer et al. [25]	2013	Rat model (retrospective)	Cytosolic and sarcoplasmatic Ca^2+^ regulation
Respress et al. [26]	2014	Rat model (retrospective)	RyR2 phosphorilation
Bederman et al. [27]	2015	Rat model (RCT)	^2^H and ^18^O in protein-bound alanine
Li et al. [28]	2019	Rat model (review)	Cardiomyocyte differentiation
Liang et al. [29]	2019	Rat model (retrospective)	NADPH oxidase/ROS
Loktev et al. [30]	2019	Rat model (RCT)	DNA methylation
Liang et al. [29]	2019	Rat model (retrospective)	MuRF1 activation
Liu et al. [31]	2020	Rat model (retrospective)	CaMKII/HDAC4 activation
Liang et al. [32]	2020	Rat model (retrospective)	Calpain system activation
Guarnieri et al. [33]	2021	H9C2 rat cardiomyocytes (retrospective)	Actin geometrical aleration
Liu et al. [34]	2023	Rat model (RCT)	DEG and DEM expression

Abbreviations: AC, adenylyl cyclase; ADP, adenosine diphosphate; ATP, adenosine triphosphate; cAMP, cyclic adenosine monophosphate; CaMKII/HDAC4, calmoduline-dependent protein kinase II/histone deacetylase 4; FN, fibronectine; GAPDH, glyceraldehyde-3-phosphate dehydrogenase; irANP, immunoreactive atrial natriuretic peptide; MDH, malate dehydrogenase; MuRF1, muscle-ring finger protein-1; NADPH, reduced nicotinamide adenine dinucleotide; NF-kB, nuclear factor kappa B; PLB, phospholamban; RCT, randomized controlled trial; RyR2, type-2 ryanodyne receptor; SERCA2a, sarcoplasmic reticulum Ca^2+^-ATPase; TM, tropomyosin.

## Data Availability

Not applicable.
